# Stable expression of shRNA for the control of recombinant adenovirus replication

**DOI:** 10.1590/1414-431X2023e12682

**Published:** 2023-07-21

**Authors:** M.V.G. Lana, F. Antunes, N.G. Tessarollo, B.E. Strauss

**Affiliations:** 1 Laboratório de Vetores Virais, Centro de Investigação Translacional em Oncologia/CTO/LIM24, Instituto do Câncer do Estado de São Paulo, Faculdade de Medicina, Universidade de São Paulo, São Paulo, SP, Brasil; 2 Laboratório de Bioinformática e Biologia Computacional, Instituto Nacional do Câncer, Ministério da Saúde, Rio de Janeiro, RJ, Brasil

**Keywords:** Adenovirus, Hexon, Iva2, Pol, shRNA, Lentivirus

## Abstract

Preventing the replication of adenovirus could have practical uses, such as controlling infection with wild-type virus or in applications involving recombinant vectors. Mainly transient methods have been used to inhibit adenovirus replication, including siRNA or drugs. Here, we tested whether stable expression of shRNA designed to target hexon, Iva2, or pol can inhibit the replication of a recombinant adenoviral vector, Ad-LacZ (serotype 5, E1/E3 deleted), in 293T cells. Significant knockdown correlating with reduced Ad-LacZ replication was achieved only when hexon was targeted. Cell sorting and isolation of cellular clones further accentuated knockdown of the hexon transcript, reduced protein levels by more than 90%, and diminished adenovirus production. As visualized by transmission electron microscopy, the cellular clone expressing the hexon-specific shRNA yielded 89.2% fewer particles compared to the parental 293T cells. Full scale production followed by purification revealed a 90.2% reduction in Ad-LacZ biological titer. These results support the notion that stable expression of shRNA can be used as a means to control adenovirus replication.

## Introduction

Adenoviruses (Ads) are non-enveloped viruses of icosahedral symmetry, having a diameter of 70 to 100 nm (1). Five different genera of Ads are known, and human adenoviruses (HAdVs) belong to the genus *Mastadenovirus*. Currently, there are seven species of HAdVs (A to G) and nearly 90 subtypes (2). HAdVs are associated with multiple pathologies in several species. HAdV infections occur mainly in children and young adults, causing, for example, respiratory and intestinal problems (3). In general, HAdV infections are mild and self-limiting. However, in immunocompromised patients, the systemic spread of the disease can cause death. In individuals with severe combined immunodeficiency (SCID), for instance, lethality rates reach over 55% (4).

Because of their importance as etiological agents, novel therapeutic approaches have been explored for the treatment of HAdV infection. Cidofovir is an acyclic nucleoside phosphate derivate of cytosine that acts as a viral DNA polymerase substrate (5). In immunocompromised patients, cidofovir is not able to prevent a fatal outcome in all cases, nor is it able to completely eliminate infections without the concomitant reestablishment of the immune system (6).

RNA interference (RNAi) is a posttranscriptional gene silencing phenomenon mediated by RNA (7). The main pathway of this process starts with the formation of a secondary structure called primary microRNA (pri-miRNA), which allows its recognition and cleavage by a microprocessor complex, forming the so-called precursor miRNA (pre-miRNA) (8). Pre-miRNA, a 60-70 nt sequence, is translocated to the cytoplasm by exportin-5 (8) where it is cleaved by the enzyme dicer, releasing a double-stranded RNA (dsRNA) sequence of 19-25 nt (7). In turn, the dsRNA binds to the RNA-induced silencing complex (RISC), which selects and incorporates one strand of this small RNA, which then can directly interact with mRNA, leading to its degradation, thus blocking translation of that sequence (7). MicroRNAs (miRNA), exogenous small interfering RNA (siRNA), artificial microRNA (amiRNA), and short hairpin RNA (shRNA) are physiological, synthetic, or recombinant examples of small RNAs that exhibit a variety of functions, from regulation of gene expression to defense against viruses (8).

Vectors for the transfer and application of RNAi, especially lentiviruses, are widely used. Among the advantages of using an expression vector is the ability to control and achieve stable, high-level expression of the shRNA of interest. Lentiviral vectors are able to transduce a wide variety of cells, whether they are undergoing cell division or not. Furthermore, they integrate into the cellular genome, allowing long-term stable expression, unlike transfected siRNAs that are lost during cell division (9). The basic structure of the shRNA expression cassette includes a promoter in conjunction with the 19 to 22 nucleotides (nt) sense sequence, a loop sequence of 3 to 10 nt followed by complementary antisense sequence, and 4 to 6 thymines that serve as a transcription terminator (10). In general, the promoters used are Pol III dependent, such as U6 and H1 (11).

The RNAi-mediated silencing of adenoviral genes has been used to successfully inhibit Ad replication. In 2007, Chung et al. (12) showed that the replication of a wild-type Ad 11 can be reduced using siRNA targeting the E1A gene, the first gene to be transcribed after virus entry into the cell, deregulating the host cell cycle. In 2010, another study used siRNA to inhibit replication of a wild-type Ad 5 by targeting E1A, IVa2, or hexon (13). IVa2 is responsible for particle assembly in the final phase of the replication cycle (14) and is also necessary for late phase-specific activation of transcription from the major late promoter and, hence, efficient production of the final mRNAs (15). Hexon is the main structural component of the viral capsid (16). Other authors have reported the successful use of siRNA or amiRNA to knockdown expression of viral DNA polymerase (pol) and pTP, genes related to viral DNA replication. Specifically, a non-replicative Ad vector was used to express amiRNA against E1A, pTP, and pol in the target cells (6,17). Another study showed effective inhibition of replication by silencing different Ad genes in combination with drugs (18). However, a stable system using RNAi to control Ad replication would be useful and has rarely been reported (19).

In this study, we investigated the use of a stable expression of shRNA to knockdown key adenoviral genes and inhibit replication of recombinant Ad vectors produced in 293T cells. Three distinct targets were evaluated in this study: hexon, IVa2 (both involved in virus assembly), and pol (necessary for replication of the viral genome). We showed here that the knockdown of hexon was particularly effective at inhibiting adenovirus multiplication.

## Material and Methods

### Cell culture and lines

The adenovirus-transformed, human embryo kidney cell line 293T (20) and the human fibrosarcoma cell line HT1080 (ATCC, CCL-121) were maintained in DMEM (Thermo Fisher Scientific, USA) supplemented with 10% FBS, 1× antibiotic-antimycotic solution (all from Thermo Fisher Scientific), at 37°C, in a humidified atmosphere of 5% CO_2_.

### Viral stocks

The first-generation adenoviral vector that encodes β-galactosidase (Ad-LacZ, Thermo Fisher Scientific) was produced as described below, titered by Adeno-X rapid titer kit (Takara Bio USA, USA), and stocked in aliquots at -80°C.

### Vector construction

The oligonucleotides encoding the shRNA sequences are shown in Supplementary Figure S1. The oligonucleotides were subjected to a T4 Kinase (New England Biolabs, USA) reaction and then annealed by heating to 95°C for 5 min and slow-cooled to room temperature. The dsDNA sequences were inserted into the lentiviral vectors (21) (kindly provided by Kristoffer Riecken, University Medical Center, Germany) LeGO 1xT/Bsd (IVa2, pol) and LeGO G/Neo-opt (hexon) that were previously digested with XhoI and HpaI. The scrambled (SCR) and galectin-3 (Gal3) shRNAs, used as controls, were kindly provided by Roger Chammas (ICESP-FMUSP, Brazil) and have been described previously (22).

### Preparation of lentivirus

The plasmid transfer vectors Lego anti-IVa2, Lego anti-pol, and Lego anti-hexon were used for lentivirus production. For each transfer vector, 10 µg was co-transfected in 293T cells along with the packaging vectors pMDL-gag/pol (6.5 µg), pRSV-Rev (2.5 µg), and pCMV-VSVg (3.5 µg) (kindly provided by Nadav Ahituv, Lawrence Berkeley Laboratory, USA). The transfection protocol was previously described (23,24). The viral supernatant was collected 24 h post-transfection and centrifuged at 660 *g* for 5 min at 4°C to reduce cellular debris. Then aliquots were stocked at -80°C.

### Production of shRNA expressing 293T cells

One hundred thousand 293T cells were seeded onto 6-well plates and transduced with 500 µL of the lentivirus supernatant in the presence of 8 µg/mL polybrene (hexamdimethrine bromide, Sigma Aldrich, USA). For the IVa2 and pol shRNA constructs, selection with 10 µg/mL blasticidin was performed. After death of control cells, the selected cells were treated with half antibiotic concentration for one week before being expanded and cryopreserved. The cells were analyzed by flow cytometry (FACSCalibur™, Becton Dickinson, USA) for dTomato reporter gene expression. For the hexon shRNA construct, selection with neomycin was not possible since the 293T cells are already resistant (20). Instead, the nearly 100% transduction efficiency was verified by detection of eGFP (enhanced green fluorescent protein) expression by flow cytometry (Supplementary Figure S2).

### Infection assays

The cell line indicated in each experiment was seeded at 5×10^5^ cells per 35-mm dish and infected the following day using a multiplicity of infection (MOI) of 1 of Ad-LacZ virus preparation diluted in culture medium to a final volume of 500 μL. The transduction was carried out at 37°C for 4 h before adding 1.5 mL of fresh medium. The cells were then cultured at 37°C for 12 to 72 h before harvesting for assays where HT1080 cells were transduced with equal volumes of adenovirus-containing cell lysate and later stained with X-gal (5-bromo-4-chloro-3-indolyl-β-D-galactopyranoside, Sigma Aldrich) or cell lysate was incubated with O-nitrophenyl-beta-D-galactopyranoside (ONPG, Sigma Aldrich), both protocols as described previously (25).

### Reverse transcription - quantitative PCR

The cell line indicated in each experiment was infected with Ad-LacZ, as described above, and harvested after 24, 36, and/or 60 h of incubation. Cells were collected with 500 µL of Trizol reagent (Thermo Fisher Scientific) using a cell scraper. Total RNA was extracted following the manufacturer's instructions and RNA concentration was determined by measuring absorbance at 260 nm. Extracted RNA quality was assessed by absorbance at 260/280 nm and by visualizing the 18 and 28S ribosomal RNA bands in agarose gel (1%). Primers used were described previously (13,17). Ribosomal 18S RNA was used as the reference gene. Total RNA (2 µg) was reverse transcribed using random primers and Moloney Murine Leukemia Virus reverse transcriptase (Thermo Fisher Scientific). Reaction conditions were 6.25 ng of cDNA (final volume of 10 µL), 4 pmol of each primer, and 5 µL of Syber Green PCR Master Mix (Thermo Fisher Scientific). Amplification conditions consisted of denaturation at 95°C for 5 min, followed by 40 cycles of denaturation at 95°C for 15 s, annealing at 60°C for 30 s, and extension at 60°C for 30 s. All samples were tested in duplicate and analyzed by the 7500 Software (Thermo Fisher Scientific). The 2^-ΔΔCt^ method was used for gene expression quantification.

### Western blotting

The cell line indicated in each experiment was infected with Ad-LacZ as described above and harvested after 36 h of incubation when total protein was extracted and quantified. For each sample, 20 µg were loaded in a 12% SDS-PAGE, separated by electrophoresis, and transferred in a nitrocellulose membrane. Mouse anti-hexon (Thermo Fisher, #LF-MA0177) and mouse anti-tubulin (EMD Millipore, #05-829, USA) monoclonal antibodies were used to probe the membrane. The Amersham ECL Prime Western Blotting Detection Reagent (GE Healthcare Lifesciences, #RPN2232, USA) was used for chemiluminescent detection in an Image Quant LAS 4000 (GE Healthcare Lifesciences).

### Electron microscopy analysis

The cell line indicated in each experiment was infected with Ad-LacZ as described above and harvested after 36 h of incubation. They were then centrifuged at 300 *g* for 5 min at room temperature and resuspended in 2% glutaraldehyde. After incubation for 2 h at room temperature, cells were centrifuged at 300 *g* for 5 min at room temperature and resuspended in phosphate-buffered saline. Inclusion with araldite resin, preparation of 50-nm ultrasections, and transmission electron microscopy (JEM-1010 JEOL, Japan) were performed by the Rede Premium core facility, FMUSP.

### Full-scale production of Ad-LacZ

For each of the 293T and 293T Clone 2 cell lines, 6×10^6^ cells/dish were seeded in twenty-five 150 mm dishes. The following day, cells were infected with MOI of 1 of Ad-LacZ. After 60 h, cells were harvested and centrifuged at 660 *g* for 5 min at 4°C. Cells were subject to three cycles of freezing/thawing and then purified by iodixanol gradient ultracentrifugation as described previously (26).

### Statistical analysis

Results were considered significant when P<0.05 using one-way or two-way ANOVA with Bonferroni post-test or Student's *t*-test. Statistical analysis was performed using Prism v.8 (GraphPad Software, USA). The number of biological and technical replicates is indicated in the figure legends.

## Results

### Knockdown of adenoviral transcripts using shRNA resulted in impaired adenovirus replication

In order to generate stable cell lines harboring a component that impedes adenoviral replication, we first constructed lentiviral vectors encoding shRNA specific for adenovirus (serotype 5): hexon, IVa2, or pol (Supplementary Figure S1). The lentiviruses were then produced and used to transduce 293T cells. In the case of Lego shIVa2 and Lego shPol, the vectors support selection with blasticidin and antibiotic-resistant cell lines were generated. While the Lego shHexon vector offers the neomycin resistance gene, 293T cells are resistant to this antibiotic due to previous manipulation (20), therefore selection with this antibiotic was not possible in this case. Even so, the transduction resulted in 94% GFP-positive cells (Supplementary Figure S2). The resulting cell lines are referred to here as 293T, shIVa2, shPol, and shHexon.

**Figure 1:**
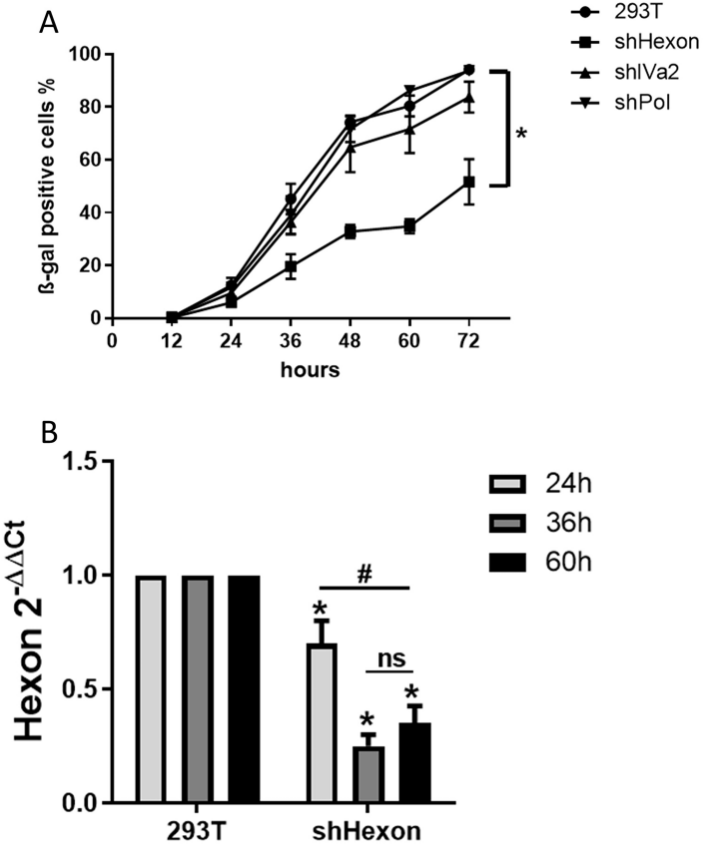
Use of shRNA to reduce virus production by hexon knockdown. **A**, Kinetics of virus production in 293T cells modified to express shRNA. The data are reported as means±SD of β-gal positive cells from 3 independent experiments. *P<0.05 (two-way ANOVA with Bonferroni post-test). **B**, Relative expression levels of adenoviral hexon targeted by shRNA in modified 293T cells upon infection with recombinant adenoviral vector Ad-LacZ. The data are reported as means±SD from 5 independent biological experiments. *P<0.05 *vs* 293T; ^#^P<0.05 24 h *vs* 36/60 h (two-way ANOVA with Bonferroni post-test).

The production of adenovirus by the shRNA-expressing cell lines was assessed. For this, the cell lines were infected with Ad-LacZ, and virus-containing lysate was collected at 12-h intervals up to 72 h post-infection. These lysates were then assayed by transducing HT1080 human fibrosarcoma cells, staining with X-gal, and counting the blue β-galactosidase-positive cells. As shown in Figure 1, the reduction in virus production was statistically significant only for the 293T shHexon cell line. These assays revealed that impairment of adenoviral multiplication by using cell lines that stably express shRNA is feasible. Since the shRNA for hexon was the most effective, the shHexon cell line was chosen for further study.

To determine if the shRNAs were indeed associated with knockdown of adenoviral transcripts, each cell line was infected with Ad-LacZ and the total RNA was collected and analyzed by RT-qPCR. A statistically significant reduction in the hexon transcript was seen at the 48-h time point (Figure 1). The IVa2 transcript levels were also diminished at 48 h while inhibition of pol did not reach significance (Supplementary Figure S3). Since only the shRNA targeting hexon was associated with both reduced transcript and virus production, we chose to focus on this construct in the remaining assays.

### Sorting and isolation of cellular clones improved hexon knockdown and further reduced adenovirus multiplication

Since transduction with lentivirus may not be homogeneous in the cell population, we postulated that cell sorting and/or isolation of cellular clones may produce lines that are especially adept for inhibition of adenovirus multiplication. The shHexon cell line was first subjected to sorting using the lentivirus-encoded eGFP as a marker, resulting in improved reporter gene expression (Supplementary Figure S2). The new Post-Sorting cell line seemed to be slightly more efficient for the inhibition of adenovirus multiplication (Supplementary Figure S4). Encouraged by this observation, we speculated that individual cell clones may be present in the mixed population that would offer even better control over adenovirus replication. Several cell clones were thus isolated by limiting dilution.

**Figure 2:**
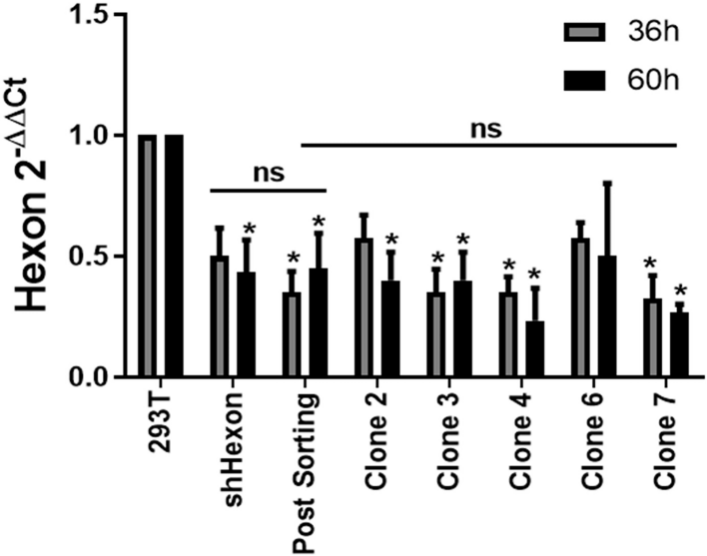
Cell sorting and isolation of cellular clones improves hexon knockdown as determined by RT-qPCR. The data are reported as means±SD from 4 independent biological experiments. *P<0.05 *vs* 293T (two-way ANOVA with Bonferroni post-test). ns: not significant.

**Figure 3:**
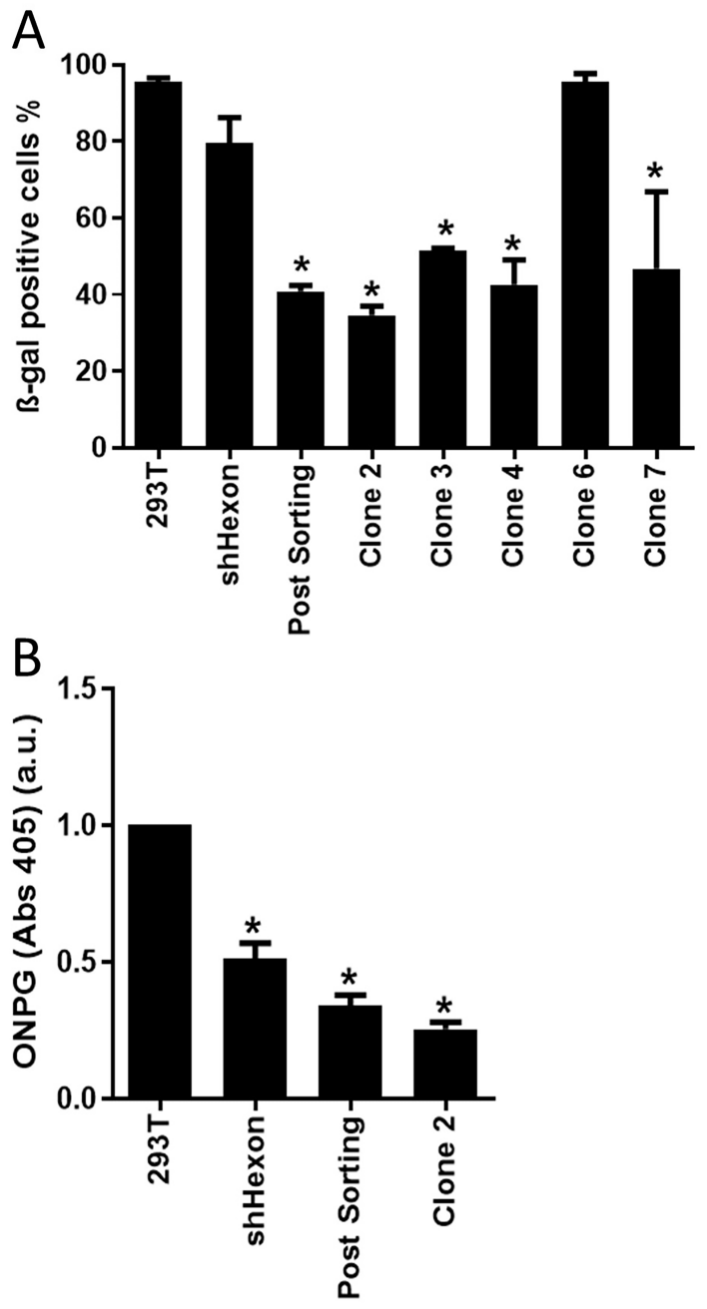
Reduced adenovirus production in cell clones with hexon knockdown. Cell lines were infected with recombinant adenoviral vector Ad-LacZ, MOI of 1, and virus-containing lysate was collected 60 h later. An equal volume of each lysate was used to transduce 1×10^5^ HT1080 cells and 48 h later these were (**A**) fixed and stained with X-gal or (**B**) incubated in the presence of the colorimetric substrate, O-nitrophenyl-beta-D-galactopyranoside (ONPG). The data are reported as means±SD of 3 independent experiments. *P<0.05 *vs* 293T (one-way ANOVA with Bonferroni post-test). a.u.: arbitrary units.

As shown in Figure 2, upon infection with Ad-LacZ, the Post-Sorting cell line showed improved knockdown of hexon transcript compared to parental cells. A similar result was also seen for several cell clones. We then used a functional assay to reveal the decrease in adenovirus production in the collection of hexon-targeting cell lines. Figure 3A shows that clone 2 was the most efficient for reducing adenovirus multiplication. We considered that the functional assay, rather than measure of knockdown, was a better indicator of the ability to impede adenovirus production. We also confirmed that reporter gene activity when measured in a colorimetric assay revealed that Clone 2 was superior to shHexon and Post-Sorting for inhibition of virus production (Figure 3B), thus Clone 2 was chosen for further study.

**Figure 4:**
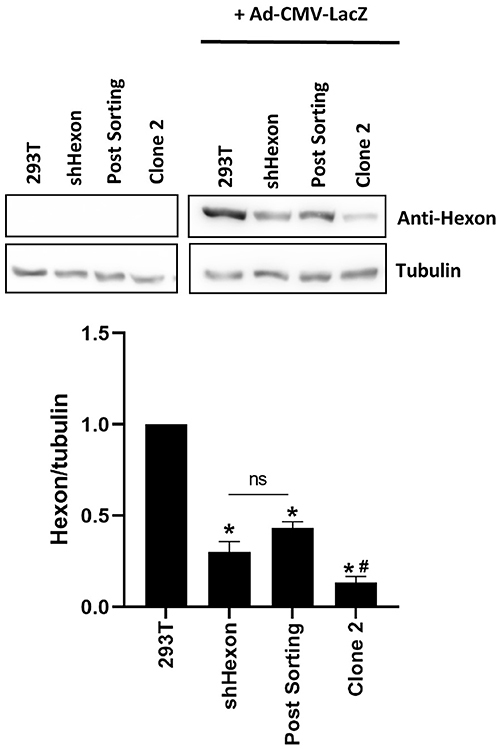
Hexon knockdown conferred reduced protein expression. Cell lines were infected or not with recombinant adenoviral vector Ad-LacZ, MOI of 1, and 36 h later total protein was extracted and subjected to western blotting. An example from three independent assays as well as quantification of the hexon band relative to tubulin are shown. *P<0.05 *vs* 293T; ^#^P<0.05 *vs* shHexon or Post Sorting (one-way ANOVA with Bonferroni post-test). ns: not significant.

As further confirmation of our stable knockdown approach, Figure 4 shows the reduction of hexon at the protein level, where Clone 2 was more efficient compared to the shHexon or Post-Sorting cell lines. We also verified that irrelevant shRNA sequences, scramble (shSCR) or targeting Gal3 (shGal3), did not impact virus production or expression of hexon protein (Supplementary Figure S5). We also show that the Clone 2 cell line proliferated at the same rate as the parental 293T cells (Supplementary Figure S6).

### Quantification of adenovirus production in Clone 2 cells

**Figure 5:**
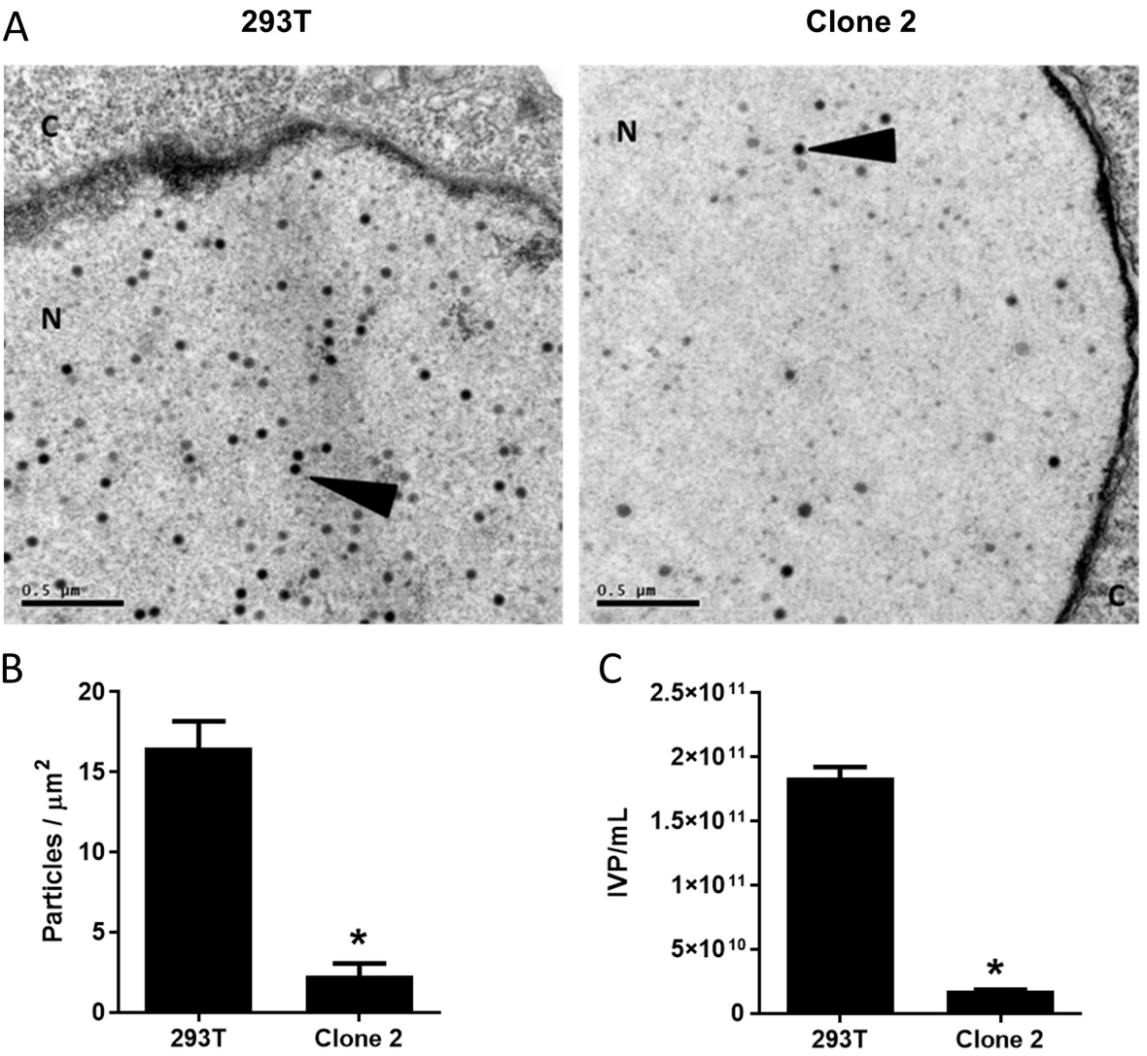
Reduced adenovirus production upon hexon knockdown. **A**, Transmission electron microscopy (scale bar 0.5 μm). For each cell line, 5x10^5^ cells were infected with recombinant adenoviral vector Ad-LacZ, MOI of 1, and 36 h later fixed for electron microscopy. In the electron photomicrographs, C indicates cytoplasm, N indicates nucleus, and arrowheads indicate adenovirus particles. **B**, Quantification of virus particles seen in 5 fields from each of 8 virus-positive nuclei were counted in two independent biological experiments and data are reported as means±SD. *P<0.05 *vs* 293T (Student's *t*-test). **C**, Quantification of infectious virus particles after full scale production and purification. IVP: infectious virus particles. *P<0.05 *vs* 293T (Student's *t*-test).

To eliminate possible biases from experimental procedures that may influence the quantification of virus production, transmission electron microscopy was performed to visualize viruses produced in the 293T and Clone 2 cells. As shown in Figure 5A, the shRNA-expressing cell line seems to harbor few virus particles compared to the parental cell line when each was infected with Ad-LacZ, MOI of 1, and processed 36 h later. All clearly visible virus particles were counted, even if their apparent density varied. By counting virus-positive nuclei, the presence of particles was quantified, revealing an 89.2% reduction when using Clone 2 cells (Figure 5B). Subtracting low-density particles from the total count did not alter the interpretation of these results (data not shown).

Finally, full-scale virus production followed by purification was performed using the 293T and Clone 2 cell lines and the biological titer was then determined. As shown in Figure 5C, the production using the hexon-specific shRNA-expressing cell line yielded 1.79×10^10^ IVP (infectious virus particles)/mL while the parental cells yielded 1.84×10^11^ IVP/mL. The shRNA approach resulted in a 90.2% reduction in virus production.

## Discussion

We showed that stable expression of shRNA targeting the adenoviral transcript for hexon can impede recombinant virus multiplication. In these assays, we demonstrated a significant reduction in hexon transcript and protein levels as well as decreased production of infectious virus as determined by small scale and full-scale production methods. Visualization of virus particles by transmission electron microscopy also revealed a reduced number of particles in the Clone 2 cell line. The use of shRNA to control adenovirus replication may have applications in the gene therapy/gene transfer arena.

Our results using shRNA showed a similar magnitude of replication inhibition compared with siRNA, as reported in the literature. In contrast, Pozzuto et al. (18) observed synergistic effects when using an engineered soluble CAR receptor and cidofovir. However, we measured inhibition by infectious titer while they examined viral genome copies to quantify inhibition. In the amiRNA study, Ibrišimović et al. revealed superior results when using pTP as a target to block wild type Ad replication in A549 cells (6). Using 293T cells, which harbor the E1A gene, may have artificially facilitated replication and confounded the efficiency of our shRNA approach. Our results are the first to reveal Ad inhibition by RNAi in a full-scale production/replication.

Although we found a 90% reduction in virus production, as seen by electron microscopy and full-scale virus production, there is still room for improvement. The literature suggests that combined siRNAs do not bring added/synergistic benefit in reducing Ad replication (6,13,17,18). Among the targets tested here, only hexon was effective, therefore no basis for its combination with other shRNA was established. Although the use of cidofovir could be an alternative, it is relatively non-specific, exhibiting varied effects on the replication or infectivity of a variety of viruses. The specificity and stability of shRNA seems to be an advantage, but further refinement of the shRNA design could potentially result in even better inhibition of hexon and thus of adenoviral multiplication.

As shown here, virus multiplication was inhibited when viral transcript encoding hexon was targeted by shRNA. In theory, the expression of a transgene should be preserved since it would typically be under the control of a heterologous promoter, and thus is independent from other viral activities, such as replication. For example, recombinant adenoviral vectors encoding single-chain antibodies, humanized monoclonal antibodies, or other therapeutic proteins could be handled using traditional methods and used to inoculate a modified cell line expressing the anti-hexon shRNA. In this scenario, purification of the recombinant protein product should be facilitated by the reduction in contaminating viral particles. Alternatively, conditionally replicating oncolytic adenoviral vectors may be targeted by this shRNA approach in order to restrict multiplication in off-target cells. For example, mesenchymal stem cells used for delivery of adenovirus, including replication competent oncolytic vectors (27,28), would be protected from viral activity.

The use of HEK293 or its derivatives may provide several potential benefits. These cells offer very high protein expression and are extensively used in industry. Subtypes that grow in suspension and without serum are readily available, and GMP certified lines already exist (29,30). The use of the 293T subtype will facilitate assays where transfection and adenovirus transduction are desired.

As shown here, the use of shRNA when stably expressed in 293T cells can provide significant reduction in recombinant adenovirus multiplication. We observed that knockdown of hexon was especially efficient and associated with a 90% decrease in virus production. Blocking viral replication while permitting transgene expression may have applications for the production of recombinant proteins or for gene therapy, such as directing the activity of oncolytic vectors.

## Supplementary Material

Click to view [pdf].
